# Current knowledge on enzymatic PET degradation and its possible application to waste stream management and other fields

**DOI:** 10.1007/s00253-019-09717-y

**Published:** 2019-04-08

**Authors:** Fusako Kawai, Takeshi Kawabata, Masayuki Oda

**Affiliations:** 1 Center for Fiber and Textile Science, Kyoto Institute of Technology, 1 Hashigami-cho, Matsugasaki, Sakyo-ku, Kyoto, 606-8585 Japan; 2 Institute for Protein Research, Osaka University, 3-2 Yamadaoka, Suita, Osaka, 565-0871 Japan; 3 Graduate School of Life and Environmental Sciences, Kyoto Prefectural University, 1-5 Hangi-cho, Shimogamo, Sakyo-ku, Kyoto, 606-8522 Japan; 4 Present address: Emeritus Professor, Okayama University, Okayama, Japan

**Keywords:** PET hydrolase, Cutinase, PETase, Structural analyses, Catalytic mechanism, Potential application

## Abstract

Enzymatic hydrolysis of polyethylene terephthalate (PET) has been the subject of extensive previous research that can be grouped into two categories, viz. enzymatic surface modification of polyester fibers and management of PET waste by enzymatic hydrolysis. Different enzymes with rather specific properties are required for these two processes. Enzymatic surface modification is possible with several hydrolases, such as lipases, carboxylesterases, cutinases, and proteases. These enzymes should be designated as *PET surface–modifying enzymes* and should not degrade the building blocks of PET but should hydrolyze the surface polymer chain so that the intensity of PET is not weakened. Conversely, management of PET waste requires substantial degradation of the building blocks of PET; therefore, only a limited number of cutinases have been recognized as *PET hydrolases* since the first PET hydrolase was discovered by Müller et al. (*Macromol Rapid Commun* 26:1400–1405, 2005). Here, we introduce current knowledge on enzymatic degradation of PET with a focus on the key class of enzymes, PET hydrolases, pertaining to the definition of enzymatic requirements for PET hydrolysis, structural analyses of PET hydrolases, and the reaction mechanisms. This review gives a deep insight into the structural basis and dynamics of PET hydrolases based on the recent progress in X-ray crystallography. Based on the knowledge accumulated to date, we discuss the potential for PET hydrolysis applications, such as in designing waste stream management.

**Electronic supplementary material:**

The online version of this article (10.1007/s00253-019-09717-y) contains supplementary material, which is available to authorized users.

## Introduction

Since the 1990s, research on the biodegradation of synthetic polymers has been promoted extensively owing to the universal concern about the global environmental crisis. Accordingly, production of biodegradable polymers has been gradually increasing, but it still comprises a low percentage of total plastic production. However, a paradigm shift from oil-based production to bio-based production has increased the interest in bio-based production of existing monomers that are currently produced from oil. However, plastic usage and the resultant plastic disposal have been increasing consistently with the increase in world population and in gross national product in developing countries. Although efforts have been made in many countries to replace oil-based plastics with biodegradable/bio-based plastics and to promote recycling of used plastics, the majority of plastic waste is dumped on land and in oceans. Plastics discarded on land are exposed to oxygen and strong sunlight, resulting in partial deterioration and cracking into pieces and finally resulting in the formation of microplastics that cause serious problems, particularly in the marine ecosystem (Moore 2008). In addition to the microplastics found in the environment, plastic microbeads are used in household products, such as toothpastes and cosmetics. Laundry produces microplastics from fiber products, and these finally enter the oceans through the sewage system (Browne et al. 2011). It is very difficult to define microplastics by size as they include tiny pieces of plastics of various shapes, such as net, ropes, microbeads, and fibers. Microplastics have been defined as plastic particles with size less than 1 mm by some scientists and as particles with size less than 5 mm by others. Most microplastics float in the water bodies and are accidently swallowed by animals, birds, and fishes as food. In the 1960s, a whale with its stomach full of plastic bags was found dead owing to starvation, and recent reports state that such dead whales are found on many seashores every year irrespective of the season. Scientists have issued an alert that microplastics are often found in the stomach of birds and fishes and in seawater and thus may be ingested by humans via the food chain. To complicate matters, plastics are hydrophobic materials that absorb chemical compounds in the environment (Teuten et al. 2009). Recently, the major cause for accidental ingestion of microplastics by animals, birds, and fishes has been reported to be their smell as food rather than their shape or size (November 9, 2018, Science Advances: http://advances.sciencemag.org/). It is, thus, a serious concern that we may ingest microplastics with environmental pollutants in the near future, a phenomenon that may have already started (https://www.theguardian.com/environment/2018/oct/22/microplastics-found-in-human-stools-for-the-first-time). There is no doubt that microorganisms play important roles in the cleanup of pollutants released by humans and in material circulation in the ecosystem, and that microbial/enzymatic degradation of polymeric materials has attracted worldwide attention since the 1990s.

Polyethylene terephthalate (PET) is one of the major plastics, in addition to polyethylene, polypropylene, polystyrene, polyvinyl chloride, and polyurethane, and its worldwide production amounted to 56 million tons in 2013 (Neufeld et al. 2016). PET has various applications, such as in manufacturing bottles, fibers, films, and containers. After use, PET bottles can be recovered through many routes, and recycling systems operate in many countries, although the overall recovery ratio is less than half and only a limited amount is recycled as new products. Currently, large amounts of PET debris are entering landfills or are left as waste, and countermeasures are needed to prevent its release in the environment and to recover used PET waste via appropriate biological, chemical, and physical treatments. Enzymatic recycling of PET has been investigated for more than 20 years, and this research is still ongoing. Although most biodegradable plastics are polyesters (e.g., polyhydroxyalkanoate, polycaprolactone, polybutylene succinate, polybutylene succinate-*co*-adipate, and poly(butylene adipate-*co*-terephthalate) (PBAT)), PET, which is also a polyester, is assumed to be recalcitrant to biodegradation. Polyesters are categorized into three groups (aliphatic, aliphatic-*co*-aromatic, and aromatic). Aliphatic polyesters (natural and synthetic) are hydrolyzed at ambient temperatures by various ester bond hydrolases. Aliphatic-*co*-aromatic polyesters are rather tough to degrade by enzymatic hydrolysis, but many probable enzymes that degrade PBAT have been identified among carboxylic ester hydrolases (EC 3.1.1), such as carboxylesterase (EC 3.1.1.1), triacylglycerol lipase (EC 3.1.1.3), and cutinases (EC 3.1.1.74). Recently, microbial enzymes belonging to arylesterase (EC 3.1.1.2) have been reported to degrade PBAT (Wallace et al. 2017). Polyesterase acting on aromatic polyesters (primarily PET) was first reported for *Thermobifida fusca* by a German research group (Müller et al. 2005). Extensive research on cutinases from *T. fusca* and other *Thermobifida* species has been performed by several groups to date. Cutinases are carboxylic ester hydrolases and first attracted attention for their phytopathogenicity; they can degrade the cutin of the cuticular layer in leaves or the suberin in bark. Research on cutinases began with phytopathogenic fungi, and the cutinase from *Fusarium solani pisi* was the most extensively characterized (Kollattukudy 1981; Heredia 2003). Subsequently, through research on polyester degradation, it was discovered that cutinases play an important role in the hydrolysis of polyesters (Tokiwa and Calabia 2007; Zimmermann and Billig 2011; Baker et al. 2012); indeed, all known PET hydrolases belong to the cutinase group. Crystal structures of PET-hydrolyzing cutinases have been studied to specify the substrate-binding domain and to introduce mutation to obtain better enzymes, as described below.

There are several excellent reviews of enzymatic PET hydrolysis and cutinases relevant to PET hydrolysis (Zimmermann and Billig 2011; Chen et al. 2013; Wei et al. 2014c; Nyyssölä 2015; Wei and Zimmermann 2017). The field of X-ray crystallography has been advancing rapidly, and several crystal structures of PET hydrolases have been solved. Here, we discuss the current knowledge on enzymatic degradation of PET while focusing on a key class of enzymes, PET hydrolases, with regard to the definition of the enzymes, requirements for PET hydrolysis, structural analyses of PET hydrolases, and their reaction mechanisms. This review gives a deep insight into the structural basis and dynamics of PET hydrolases based on the recent progress in X-ray crystallography. Based on the knowledge accumulated to date, we discuss the various applications of PET hydrolysis, such as in designing waste stream management.

## Definition of PET hydrolases and factors required for significant degradation of PET

The important mechanism of enzymatic PET degradation is surface hydrophilization of PET fibers. For this purpose, only surface modification is needed to improve factors such as finishing fastness, dyeability, wettability, and anti-pilling behavior, but degradation of the inner building block of PET is unfavorable as it weakens the fiber strength. Many enzymatic treatments have been tested using various hydrolases, such as lipases from *Candida antarctica* (Vertommen et al. 2005), *Thermomyces lanuginosus* (Eberl et al. 2009), *Burkholderia* spp. (Lee and Chung 2009), and *Triticum aestivum* (Nechwatal et al. 2006); cutinases from fungi, *Aspergillus oryzae*, *Humicola insolens* (Ronqvist et al. 2009), *Penicillium citrinum* (Liebminger et al. 2007), and *Fusarium solani* (Alisch-Mark et al. 2006; O’Neill et al. 2007); and those from actinomycetes, *T. fusca* (Brueckner et al. 2008), *Thermobifida cellulosilytica* (Herrero Acero et al. 2011), *Thermobifida alba* (Ribitsch et al. 2012b), *Saccharomonospora viridis* (Kawai et al. 2017); esterase (*Thermobifida halotolerans*, Ribitsch et al. 2012a); and carboxylesterases (Billig et al. 2010). Even a protease, papain, can improve the hydrophilicity of polyester fabrics (Kim and Song 2010). Biundo et al. (2018b) reviewed surface engineering technology of polyester-degrading enzymes.

**Fig. 1:**
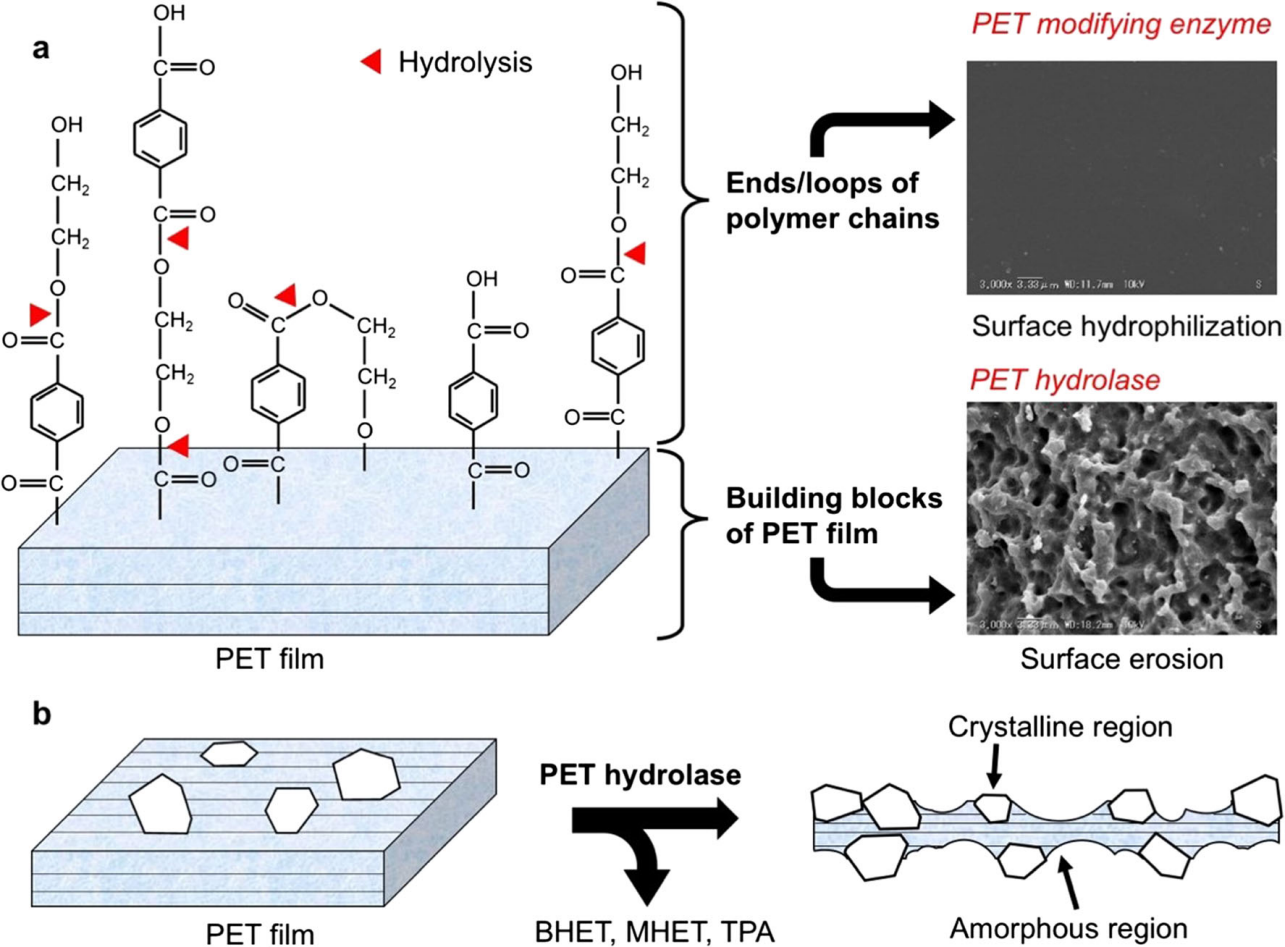
Image of PET film and its relevance to enzymatic attack. **a** PET film structure and roles of *PET-modifying enzyme* and *PET hydrolase*. Partially reproduced from Oda et al. (2018). Copyright 2018 Springer. **b** Preferential attack on amorphous region of PET film. BHET, bis(hydroxyethyl)terephthalate; MHET, mono(hydroxyethyl)terephthalate; TPA, terephthalic acid

On the surface of PET, either fiber or film, the ends of polymer chains are expected to protrude, or a part of the polymer chain may form a loop, and these are hydrolyzed to carboxylic acid and hydroxyl residues, thus increasing surface hydrophilicity (Fig. 1a). The hydrolyzable-surface PET chains are thought to comprise < 0.1% of amorphous PET film (Goodfellow Cambridge, Ltd.) (PET-GF; 0.25 mm thick; crystallinity of 6.3%) and amorphous PET film for packaging (Sanwa Supply, Inc., Okayama, Japan) (PET-S; 0.6 mm thick; crystallinity of 8.4%) (Oda et al. 2018) (Table S1). Any textile or film of PET may have a hydrolyzable polymer end or a loop on their surface (Table S1) (Kawai et al. 2017; Zimmermann and Billig 2011). Some researchers designated these modifying enzymes as PET hydrolases. However, Müller et al. (2005) first reported the apparent degradation of the inner block of PET film, wherein the degradation rate is far higher than that of surface hydrolyzable residues. Since then, several cutinases have been reported to degrade the inner block of PET film, for which commercially available PET-GF is often used and can provide a standard for the comparison of their PET hydrolysis abilities. We propose to categorize the enzymes into two groups: *PET surface–modifying enzymes* that limit the degradation rate at the surface hydrophilization level without visible change by SEM observation and *PET hydrolases* that can significantly degrade the inner block of PET (by at least 10%) with visible change by SEM observation, as shown in Fig. 1a. PET hydrolases can be used for surface hydrolysis, but PET surface–modifying enzyme cannot significantly degrade the building block of PET. Here, we focus on structural comparisons of PET hydrolases.

What factors are necessary for significant degradation of the building blocks of PET? In general, the hydrophobicity, crystallinity, surface topography, and molecular size of synthetic polymers are important factors affecting their biodegradability (Tokiwa et al. 2009; Webb et al. 2013; Wei and Zimmermann 2017). For PET hydrolysis, we must take additional factors into consideration. It is well known that the polymer chain fluctuates at temperatures higher than the glass transition temperature (*T*_g_). The *T*_g_ value of PET is approximately 80 °C, but it is lowered in water by water molecules diffusing between polymer chains, resulting in increased chain mobility (Kikkawa et al. 2004). Enzymatic reactions are performed in aqueous solutions, in which *T*_g_ values of PET are 60–65 °C (Kawai et al. 2014). PET has water absorbency of 0.1–1.0%. Water molecules enter between the polymer chains, weaken hydrogen bonds, randomize polymer chains, and increase polymer chain flexibility, which increases their accessibility to enzymes. Water absorbency must increase in water and at higher temperatures. Therefore, it is expected that higher PET hydrolase reaction temperatures will result in faster rates of PET degradation, as reported by Kawai et al. (2014) and Oda et al. (2018).

PET has different crystallinities according to its usage. Most PET used for manufacturing bottles and textiles has high crystallinity of 30–40%. However, PET used for packaging has less crystallinity, e.g., approximately 8% (Kawai et al. 2014). Commercially available low-crystallinity PET (PET-GF) has approximately 6–7% crystallinity (Ronqvist et al. 2009; Kawai et al. 2014). Polyesterases are thought to preferentially hydrolyze amorphous regions of PET (Welzel et al. 2002; Brueckner et al. 2008; Ronqvist et al. 2009; Donelli et al. 2010; Gamerith et al. 2017), as shown in Fig. 1b. Increased crystallinity limits the movement/fluctuation of polymer chains and decreases the availability of polymer chains for enzymatic attack. In addition, PET bottles and textiles are further stretched to orient the polymer chains in order, and sometimes PET is further biaxially oriented (bo); this strengthens the hydrogen bonds between polymer molecules and results in additional stretch-induced crystallization (Wei and Zimmermann 2017). The surface topology of PET materials is dependent on the crystallinity and orientation during production. PET bottles are typically formed as follows: PET pellets are melted at approximately 270 °C and subjected to injection molding. Consequently, the orientation of the polymer chain is different depending on the different parts of a bottle. Unfortunately, no PET hydrolases are known that can be applied for direct digestion of PET bottles, textiles, and biaxially stretched films. However, less crystalline PET can be significantly hydrolyzed by several cutinases, as mentioned below.

In conclusion, when we consider enzymatic PET degradation, five factors must be addressed: (1) reaction temperatures, preferably over *T*_g_; (2) water absorbency, highly related to temperature and crystallinity and orientation of polymer chains; (3) crystallinity; (4) orientation of polymer chains, in order or amorphous; and (5) surface topology, dependent on the crystallinity and orientation of polymer chains. For a given PET film, it is reasonable to consider that the higher the reaction temperature, the higher the degradation rate will be. In summary, PET hydrolysis is the interactive result of polymer chain flexibility and cutinase structure (active site accessibility to the polymer surface) (Zumstein et al. 2017).

## Biochemical bases for PET hydrolases

The possibility of enzymatic hydrolysis of PET films was first reported by Müller et al. (2005). They first purified an enzyme from the culture supernatant of *T. fusca* (known as the priority species in compost) that hydrolyzed poly(butylene terephthalate-*co*-adipate) (BTA) and determined the amino acid sequence of BTA hydrolase 1 (Kleeberg et al. 2005). Müller et al. (2005) reported that non-recombinant and recombinant hydrolases hydrolyzed two kinds of PET films by approximately 40–50% at 55 °C in 3 weeks, although detailed information of the DNA sequence was not shown. GenBank accession AJ810119.1 indicates two genes coding for BTA hydrolases 1 and 2. The genome of *T. fusca* YX was sequenced to determine the presence of two triacylglycerol lipase genes (*Tfu0882* and *Tfu0883*) (Lykidis et al. 2007) that apparently code for cutinase without a lid structure. The sequence of *Tfu0883* is consistent with that of the gene for BTA-1 (see Table 1). With regard to the activity on *p*-nitrophenol (*p*-NP) acyl ester, esterases act on short-chain acyl esters, cutinases act on short–medium-chain acyl esters (approximately up to C_8_–C_10_), and lipases act on medium–long-chain acyl esters (more than C_10_). It is noteworthy that selectivity of acyl length toward *p*-NP acyl ester is used to define carboxylesterases, but activity on *p*-NP acyl ester is not correlated to the activity of PET hydrolysis (Heumann et al. 2006; Biundo et al. 2018b). In our previous study (Kawabata et al. 2017), no correlation was found between the activities on *p*-NP butyrate and poly(butylene succinate-*co*-adipate) (PBSA) (unpublished data). A strict definition of cutinases is dependent on the hydrolysis of cutin. The cutinase activities of some polyesterases (e.g., BTA-1 and Tfu0883) were confirmed (Kleeberg et al. 2005; Chen et al. 2008), but those of some polyesterases (e.g., Cut190 and Tha_Cut1) were not analyzed (Kawai et al. 2014; Ribitsch et al. 2012b). Cutin and its components are not commercially available, and the determination of cutin hydrolysis is troublesome. However, cutin degradation is not crucial for polyesterases, although cutinase activities are important for plant pathogens (Kollattukudy 1981; Heredia 2003). In addition, X-ray crystallography of PET hydrolases and homologous enzymes (Fig. S1) apparently suggested that their structures were almost identical, irrespective of their origin. Therefore, it is reasonable to categorize all of them as cutinase members.

**Table 1:** Reported PET hydrolases and their homologs

Name	Source	GenBank accession	Sequence identity (%)	References	PDB ID	References
Lipase	*Streptomyces exfoliatus*	–	62.8	Wei et al. (1998)	1JFR	
BTA-1 (TfH)*	*Thermobifida fusca* DSM43793	AJ810119.1	100	Müller et al. (2005); Kleeberg et al. (1998, 2005); Dresler et al. (2006)		
BTA-2			92.3			
Tfu_0882	*Thermobifida fusca* YX (*T. fusca* WSH03-11)	AAZ54920.1	93.1	Lykidis et al. (2007); Chen et al. (2008); Su et al. (2013)		
Tfu_0883*		AAZ54921.1	100			
TfCut1	*Thermobifida fusca* KW3	CBY05529.1	94.3	Herrero Acero et al. (2011)		
TfCut2*		CBY05530.1	99.1		4CG1, 4CG2, 4CG3	Roth et al. (2014)
Est1*	*Thermobifida alba* AHK119	BAI99230.2	83.1	Hu et al. (2010)		
Est119		BAK48590.1	82.4	Thumarat et al. (2012)	3VIS, 3WYN6AID	Kitadokoro et al. (2012); Kawai et al. (2013); Kitadokoro et al. (2019)
Thc_Cut1	*Thermobifida cellulosilytica* DSM44535	ADV92526.1	100	Herrero Acero et al. (2011)	5LUI, 5LUJ, 5LUK, 5LUL	Ribitsch et al. (2017)
Thc_Cut2*		ADV92527.1	93.1			
Thf42_Cut1	*Thermobifida fusca* DSM44342	ADV92528.1	97.7	Herrero Acero et al. (2011)		
Tha_Cut1	*Thermobifida alba* DSM43185	ADV92525.1	98.5	Ribitsch et al. (2012b)		
Thh_Est	*Thermobifida halotolerans* DSM44931	AFA45122.1	75.1	Ribitsch et al. (2012)		
TfAXE	*Thermobifida fusca* NTU22	ADM47605.1	98.9	Huang et al. (2010)		
LC-cutinase	Metagenome from leaf-branch compost	AEV21261.1	56.6	Sulaiman et al. (2012)	4EB0	Sulaiman et al. (2014)
Cut1	*Thermobifida fusca* NRRL B-8184	JN129499.1	93.1	Hegde and Veeranki (2013)		
Cut2		JN129500.1	100			
Tcur1278	*Thermomonospora curvata* DSM43183	ACY96861.1	61.9	Chertkov et al. (2011); Wei et al. (2014b)		
Tcur0390*		ACY95991.1	61.9			
Cut190	*Saccharomonospora viridis* AHK190	AB728484	65.6	Kawai et al. (2014)		
Cut190_S226P					4WFI,4WFJ, 4WFK	Miyakawa et al. (2015)
Cut190_S226P_R228S (Cut190*)				Oda et al. (2018)	5ZNO, 5ZRQ, 5ZRR, 5ZRS	Numoto et al. (2018)
PETase	*Ideonella sakaiensis* strain 201-F6	GAP38373.1	51.7	Yoshida et al. (2016)	5XG0, 5XFY, 5XFZ, 5XH2, and 5XH3 5XJH and 5YNS 6ANE 6EQD, 6EQE, 6EQF, 6EQG, 6EQH	Han et al. (2018); Joo et al. (2018) Fecker et al. (2018); Austin et al. (2018)
Cbotu_EstA*	*Clostridium botulinum* ATCC3502	KP859619	–	–	5AH1	Perz et al. (2016); Biundo et al. (2018a)
Cbotu_EstB		KP859620				

The sequence identities were calculated based on the pairwise alignments of the mature protein sequences of each enzyme with the first described synthetic polyester hydrolase BTA-1 (TfH) from *T. fusca* DSM43793

*Higher activity than the other enzymes

Based on the genome sequence of *T. fusca* YX, Chen et al. (2008) cloned two cutinases (Tfu_882 and Tfu_883, with the activity of the latter being higher than that of the former) from a laboratory stock of *T. fusca* and reported that the amino acid sequence of Tfu_883 is identical to BTA hydrolase 1. A set of cutinases have been cloned from *T. fusca* KW3 (Herrero Acero et al. 2011) and from *T. fusca* sp. NRRL B-8184 (Hegde and Veeranki 2013). Other groups have also cloned cutinases from *T. fusca* (Table 1). Acetylxylan esterase cloned from *T. fusca* NTU22 (TfAXE) also exhibits high similarity to cutinases (Huang et al. 2010). *T. fusca* cutinases are the most researched among actinomycete cutinases, which are expressed in hosts other than *Escherichia coli*, such as *Streptomyces rimosus* (Sinsereekul et al. 2010), *Streptomyces lividans* (Li et al. 2013), *Bacillus megaterium* (Yang et al. 2007), and *Pichia pastoris* X-33 (TfAXE; Yang et al. 2010).

Hu et al. (2008) suggested the presence of thermostable polyesterases (most probably cutinases) in versatile thermophilic bacteria (primarily actinomycetes and *Bacillus* species) obtained from compost and cloned a new cutinase (Est119) from *T. alba* AHK119 (Hu et al. 2010). *T. alba* AHK119 possessed two tandem cutinase genes (*est119* (*est2*) and *est1*, with the activity of the latter being higher than that of the former) (Thumarat et al. 2015). A cutinase from *T. alba* DSM43185 was also cloned by Ribitsch et al. (2012b). On the other hand, Herrero Acero et al. (2011) cloned two cutinases from *T. cellulosilytica* DSM44535, viz. Tc_Cut1 and Tc_Cut2, the amino acid sequences of which are identical to those of Tfu_0883 and Tfu_0882, respectively, from *T. fusca* YX. Tc_Cut1 showed higher activity than Tc_Cut2, indicating the surface hydrolysis of the PET film (crystallinity of 37%) at 50 °C for 120 h. All *Thermobifida* species so far reported have indicated the presence of tandem cutinase genes, which share high homology with each other, but their activity levels differ immensely. Homologies of cutinase genes conserved in *Thermobifida* are high even among species. A possible explanation for this is that the ancestral cutinase possessing polyester degradability, most probably originating from a species of *Thermobifida*, was copied in one species and then a set of cutinase genes was transferred to other species and the same events were repeated. As the cutinase genes of *T. fusca* and *T. cellulosilytica* are almost identical, either of these species may contain the ancestral gene. Perz et al. (2016) cloned two genes coding for the esterases Cbotu_EstA and Cbotu_EstB from an anaerobic bacterium, *Clostridium botulinum* ATCC 3502, and analyzed the crystal structure of the more efficient enzyme Cbotu_EstA. Cbotu_EstA could hydrolyze PET at 50 °C only by a marginal level, but truncation of the N-terminal 71 residues exposed a hydrophobic patch on the surface, similar to a cutinase, and introduction of mutation in the zinc-binding domain improved the hydrolytic activity on PET at 50 °C (Biundo et al. 2018a). Based on the complete genome sequence of *Thermomonospora curvata*-type strain (B9) (Chertkov et al. 2011), Tcur1278 and Tcur0390 have been cloned from *T. curvata* DSM43183 (Wei et al. 2014b). *T. curvata* is a thermophilic actinomycete phylogenetically related to *T. fusca* and possesses two homologous genes coding for hydrolases; these hydrolases are most probably cutinases, although their activities on cutin have not been examined. Both enzymes shared 62% sequence identity with BTA hydrolase 1 and decomposed PET nanoparticles (no crystallinity), but it is questionable whether they can significantly degrade PET film, e.g., PET-GF, as their thermostability is at most 50 °C for Tcur1278 and < 50 °C for Tcur0390 for 60 min. A temperature higher than 60 °C is required for significant degradation of PET-GF (Zimmermann and Billig 2011; Kawai et al. 2014; Oda et al. 2018). An esterase cloned from *T. halotolerans* DSM44931 has high homology with actinomycete cutinases and can hydrophilize PET film at 50 °C (Ribitsch et al. 2012a), although no information was given about the origin of the PET film and its properties, such as crystallinity. Kawai et al. (2014) cloned a cutinase-like enzyme (Cut190) from *S. viridis* AHK190 isolated from compost. Sulaiman et al. (2012) cloned a cutinase (LC-cutinase) based on the metagenomic library from leaf-branch compost, which is considered to be a mixed gene pool of cutinase genes from compost actinomycetes. It can be concluded that thermophilic actinomycetes are the most promising microbial sources of PET hydrolases. Table 1 summarizes the recombinant cutinases and homologous enzymes, including cutinases from *T. fusca* (Müller et al. 2005; Then et al. 2015; Wei et al. 2014c) and *S. viridis* (Kawai et al. 2014), and metagenomes (Sulaiman et al. 2014) corresponding to PET hydrolases, as defined above.

Ronqvist et al. (2009) compared the catalytic activities of cutinases from *H. insolens* (HiC), *Pseudomonas mendocina*, and *F. solani* using low-crystallinity (lc) and bo PET films (both are commercial products from Goodfellow Cambridge, Ltd.; lcPET is the same as PET-GF). HiC degrades lcPET tremendously at 70 °C, but other cutinases showed far lower degradability at 50 °C and 40 °C, as their thermostability is less than 50 °C. The degradation rate of HiC at 70 °C reached approximately 100% in 96 h, but under the same reaction conditions with HiC, no measurable weight loss of boPET was observed. The crystallinity of lcPET increased from 7.0 ± 0.5 to 9% under control conditions heated without an enzyme at 70 °C for 96 h, and the cold crystallization peak shifted to a lower temperature compared to the initial sample, a phenomenon known to occur for even small changes in crystallinity (Pingping and Dezhu 1997). HiC-treated film did not exhibit a distinct crystallization peak, suggesting a drastic change in the polymer structure. In aqueous solutions at 70 °C, lcPET film must increase water absorbency, loosen orientation of polymer chains, and increase accessibility of polymer chains to the enzyme. In addition, degradation of an amorphous region may affect a crystalline region, finally resulting in complete degradation. HiC was provided by Novozymes (Bagsvaerd, Denmark), and its exact sequence information and structural analysis are unavailable, although some crystal structures of cutinases from *H. insolens* have been obtained from the Protein Data Bank (PDB) and used for comparison with other cutinases, as described below.

Yoshida et al. (2016) isolated *Ideonella sakaiensis* proliferating on an amorphous PET film (1.9% crystallinity) at 30 °C for 40 days and cloned a gene for PETase. PETase shares 45–53% amino acid sequence identity with the actinomycete cutinases (Fig. S2). However, the degradation rate of the PET film was too low for the degradation of the inner block of PET (not mentioned in the study but can be calculated to be at most less than 1%) (Table S2), and the enzyme is heat-labile and therefore lacks the requisite features to be defined as a PET hydrolase, as described above. When we incubated two kinds of amorphous films and PET microfiber with a Cut190* mutant (Cut190*/Q138A/D250C-E296C, where Cut190* is Cut190/S226P/R228S) at 30 °C, the total products amounted to approximately 0.01–0.03% of the weights of two films in 22 h and were almost constant thereafter (Table S1). However, PET microfiber (crystallinity of 14.1%) was significantly hydrolyzed to approximately 25% with increased time, although the microfiber has much higher crystallinity. The difference between the hydrolysis rates of the films and microfiber is thought to be dependent on the surface dimension and orientation/surface topology of PET. As the same microfiber is completely degraded by Cut190*/Q138A at 65 °C in 15 h (Oda et al. 2018), the crystalline part must be susceptible to enzymatic attack. The results obtained with HiC also indicated that the crystalline part is attacked (Ronqvist et al. 2009). We suggest that the amorphous region is attacked first, which must make the crystalline region susceptible to enzymatic attack, and finally both the amorphous and crystalline regions are digested. The polymer chains must become sufficiently flexible to be susceptible to enzymatic attack. A microfiber has no orientation of polymer chains, which is most likely a major cause for the enzymatic attack at 30 °C, but polymer chains of PET-GF and PET-S become sufficiently flexible for enzymatic attack at 60–65 °C. Therefore, whether PETase can be a PET hydrolase and whether PETase can hydrolyze PET-GF at 30 °C still remain to be determined.

PETase has been crystallized independently by several groups (Han et al. 2018; Joo et al. 2018; Liu et al. 2018; Fecker et al. 2018; Austin et al. 2018) to investigate its ability to degrade PET. The crystal structure of PETase and its sequence homology with cutinases apparently indicated that PETase is a member of the cutinases. As none of these studies on PETase used PET-GF film, as often used by other researchers (Ronqvist et al. 2009; Kawai et al. 2014; Then et al. 2015), it is extremely difficult to compare their results with previous reports and to evaluate their exact ability, but classification of PETase as a PET hydrolase is questionable. The physical properties of a film, the crystallinity and orientation of polymer chains and the surface topology, must severely affect enzyme attack and the resultant degradability.

In summary, there is no doubt that all effective PET hydrolases and even PBAT-degrading enzymes are cutinases and homologous enzymes. The unique properties of cutinases are dependent on their protein structures that lack a lid covering the active site, although lids are found to cause interfacial activation in lipases. Cutinases generally possess shallow, open active sites that are exposed to the solvent. These reasons can explain why PET hydrolases are limited to cutinases, although surface modification of PET is possible with lipases, esterases, and cutinases. Esterases are defined as exhibiting higher activity on short-chain acyl esters of *p*-NP (Bornscheuer 2002), suggesting that they may not recognize hydrophobic PET. However, cutinases act on longer acyl esters of *p*-NP than esterases and on hydrophobic cutin (Chen et al. 2013). Cutinases were first studied from pathogenic fungi, typically *F. solani pisi* that utilizes a cutinase to invade the cuticular layer of plants (Kollattukudy 1981; Heredia 2003). The name cutinase is derived from cutin existing in the cuticular layer of plants. Non-pathogenic fungi, such as *P. citrinum* (Liebminger et al. 2007) and *H. insolens* (Ronqvist et al. 2009), also produce cutinases. Except for HiC, no fungal PET hydrolases have been documented to date.

**Fig. 2:**
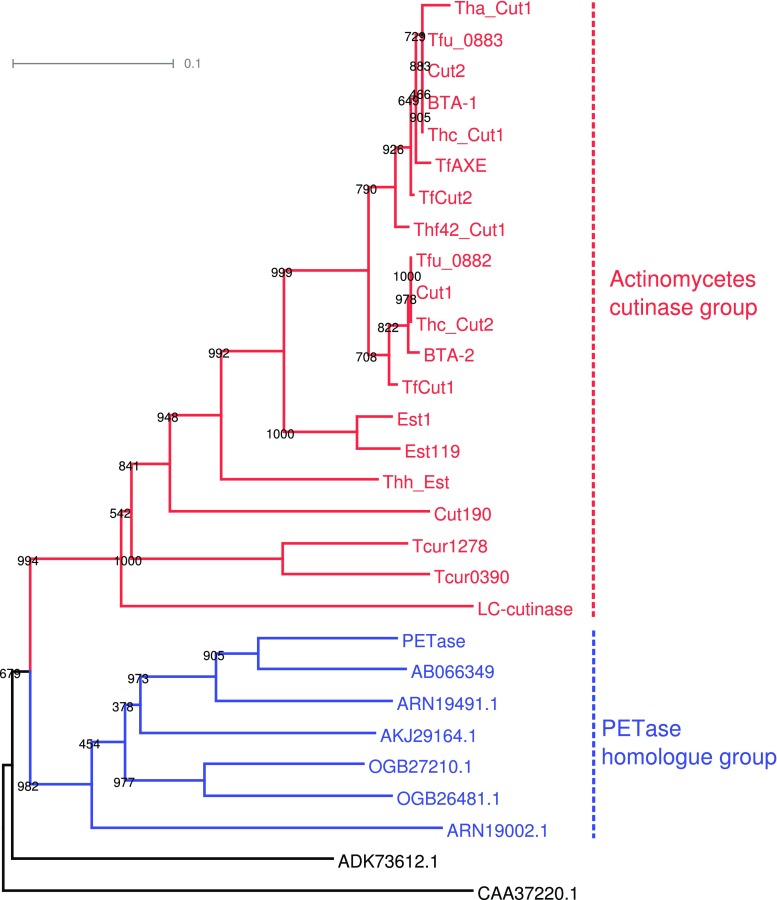
Phylogenetic tree for amino acid sequences of the reported PET hydrolases and their homologs. A multiple alignment and a tree were constructed using the program ClustalW2 (Larkin et al. 2007). The tree was generated by the neighbor-joining algorithm using sites without any gaps and was displayed as a midpoint-rooted tree using the program Dendroscape 3 (Huson and Scornavacca 2012). Numbers on the ancestor nodes are bootstrap values calculated by 1000 bootstrap samples. Actinomycete cutinases are represented using the enzyme names shown in Table 1. Six additional homologs for PETase were taken from the UniProt/TrEMBL database. GenBank accession AB066349 is poly(tetramethylene succinate) depolymerase from *Acidovorax delafieldii* (Uchida et al. 2002). The other five accession IDs are uncharacterized proteins found in bacteria as follows: ARN19491.1 and ARN19002.1 are from *Rhizobacter gummiphilus*, OGB27210.1 and OGB26481.1 are from *Burkholderiales* bacterium, and AKJ29164.1 is from *Polyangium brachysporum*. Two remote bacterial homologs (ADK73612.1 and CAA37220.1) were included to determine the root. ADK73612.1 is cutinase A from *Pseudomonas pseudoalcaligenes*, and CAA37220.1 is lipase 1 from *Moraxella* sp. (strain TA144)

Cutinases were grouped into fungal cutinases, including those from phytopathogenic and non-phytopathogenic fungi and bacterial cutinases, including those from actinomycetes and others. PETase has a sequence identity of more than 65% with many hydrolases from non-thermophilic bacteria (their ability to degrade PET has not been reported), among which poly(tetramethylene succinate) depolymerase (accession number AB066349) from *Acidovorax delafieldii* strain BS-3 (Uchida et al. 2002) showed the highest sequence identity of 82%. The phylogenetic tree of bacterial cutinases indicated that PETase formed a group with hydrolases from non-thermophilic bacteria and actinomycete cutinases formed a separate group (Fig. 2).

## Structural analyses of cutinases by X-ray crystallography

**Fig. 3:**
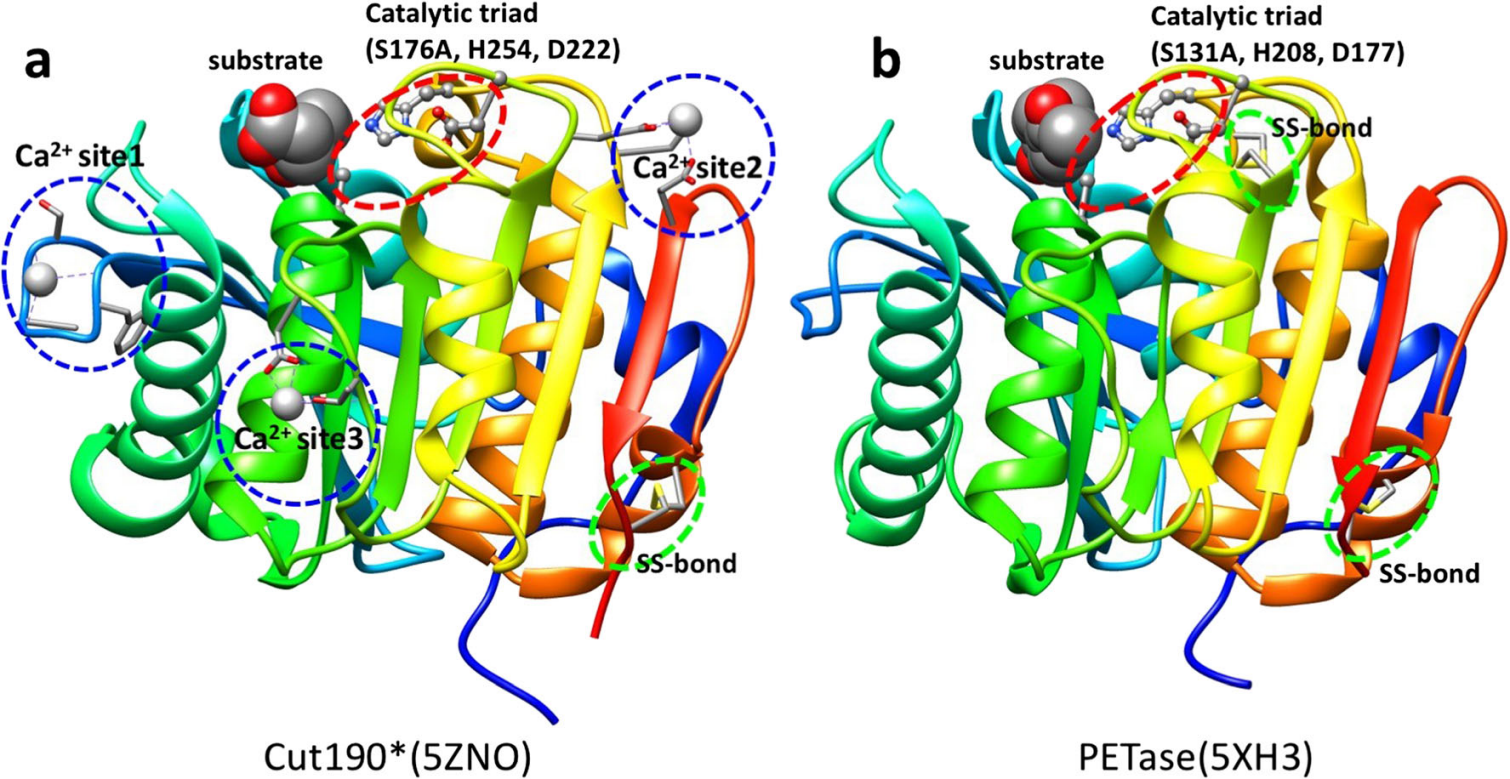
Comparison of overall 3D structures of Cut190* and PETase. The main chains are shown in ribbon models, the side chains of catalytic triads are shown in ball and stick models, and Ca^2+^ binding sites and SS bonds are shown in stick models. Substrates are shown in space-filling models. Note that serine in the catalytic triad (S176 or S131) is replaced with alanine. **a** Cut190 (PDB ID: 5ZNO), which has three Ca^2+^ binding sites and one SS bond. The bound substrate (ethyl succinate) is taken from PDB ID: 5ZRR. **b** PETase (PDB ID: 5XH3), which has no Ca^2+^ binding site and two SS bonds. The bound substrate is 1-(2-hydroxyethyl) 4-methyl terephthalate. Molecular graphics were generated using UCSF Chimera (Pettersen et al. 2004)

Cutinases first attracted attention for their role in phytopathogenicity. The crystal structure of a cutinase from the phytopathogenic fungus (*F. solani pisi*) has been well studied (Martinez et al. 1992; Longhi et al. 1997a, b). Subsequently, many aliphatic polyester–assimilating microorganisms were isolated, and their polyesterases were characterized as cutinases (Tokiwa and Calabia 2007; Zimmermann and Billig 2011; Baker et al. 2012). Following the discovery of PET hydrolase by Müller et al. (2005), several groups have cloned aromatic polyesterase genes from thermophilic actinomycetes, as described above. Some polyesterases are capable of cutin hydrolysis and their crystal structures are essentially identical to each other, suggesting that aromatic polyesterases belong to the cutinase group. The first crystallization of an actinomycete cutinase was reported for Est119 from *T. alba* AHK119 (Kitadokoro et al. 2012). Est119 was analyzed as an alpha/beta hydrolase with nine beta strands surrounded by eight alpha helices, based on the crystal structure of a homologous lipase from *Streptomyces exfoliatus,* (PDB ID: 1JFR) that is apparently a cutinase-like enzyme with no lid (Wei et al. 1998). Subsequently, the crystal structures of several cutinases from actinomycetes were analyzed by X-ray crystallography (Table 1), viz. LC-cutinase from a metagenome (Sulaiman et al. 2014), TfCut2 from *T. fusca* KW3 (Roth et al. 2014), Cut190 from *S. viridis* AHK190 (Miyakawa et al. 2015; Numoto et al. 2018), and Thc_Cut1 from *T. cellulosilytica* (Ribitsch et al. 2017), and these shared approximately the same size and structure (Fig. S1). Miyakawa et al. (2015) determined the active (Ca^2+^-bound) and inactive (Ca^2+^-unbound) forms of Cut190_S226P. This is the only reported cutinase for which the inactive form occurs in the absence of Ca^2+^; other homologous cutinases always retain the active forms. The active form of cutinases is similar to each other, including the active form of Cut190 (Oda et al. 2018). The crystal structures of Cut190* complexed with Ca^2+^/Zn^2+^ and model substrates (monoethyl succinate and monoethyl adipate) have been solved (PDB IDs: 5ZNO, 5ZRQ, 5ZRR, and 5ZRS) (Numoto et al. 2018) (Fig. 3a). Roth et al. (2014) analyzed the crystal structure of TfCut2 complexed with a competitive inhibitor, phenylmethanesulfonic acid (PDB ID: 4CG2).

As described above, it is questionable whether PETase can significantly degrade PET-GF film because the reported significant degradation of this film was performed at 65–70 °C, although the amino acid sequence of PETase is similar to those of actinomycete cutinases. Several research groups have been successful in crystallizing PETase (Han et al. 2018; Liu et al. 2018; Joo et al. 2018; Fecker et al. 2018; Austin et al. 2018). Han et al. (2018) obtained the crystal structure of a PETase mutant complexed with a PET model compound, 1-(2-hydroxyethyl) 4-methyl terephthalate (HEMT) (Fig. 3b), and reported that the additional disulfide bond besides the disulfide bond commonly found in cutinases is unique to PETase (Fig. 3b and Fig. S2), but PETase is heat-labile and its degradation ability is very low even for PET film with a very low crystallinity. Actinomycete cutinases have only one disulfide bond (Fig. 3a), but their thermostability is sufficient for degradation of PET-GF. We introduced one more disulfide bond in Ca^2+^ binding site 2 of Cut190* (Fig. 3a and Fig. S2) and confirmed the remarkable raise of *T*_m_ value (Oda et al. 2018). The resultant overall structure of PETase overlapped well with those of actinomycete cutinases, but the enzyme had a broader, open active-site cleft than those in homologous cutinases (Joo et al. 2018; Austin et al. 2018; Chen et al. 2018). Austin et al. (2018) reported that narrowing the binding cleft via mutation of two amino acids in the active site to conserve amino acids among homologous cutinases improves PET degradation, suggesting that PETase is not fully optimized for PET degradation. Chen et al. (2018) argued against Austin et al. (2018), stating that “W156 wobbling” is different from that in other cutinases, although amino acid residues in the substrate-binding pocket are conserved or semi-conserved in PETase and homologous cutinases. In addition, S185, which was mutated to S185H by Austin et al. (2018), is a critical residue that endows W156 with its flexibility.

Very recently, Kitadokoro et al. (2019) solved the crystal structure of Est119 complexed with ethyl lactate (PDB ID: 6AID). The overall structures of the cutinase–substrate complexes are almost identical, but slight variations are observed in the loop regions. Therefore, irrespective of their inability to degrade the building blocks of PET, these cutinases share the same structures as those of PET hydrolases. It is noteworthy that a specific substrate-binding domain is absent in these cutinases, unlike polyhydroxyalkanoate depolymerase (Knoll et al. 2009) and cellulase (Atthoff and Hilborn 2007). The hydrophobic regions surrounding the catalytic site most probably contribute to absorption on the PET surface (Herrero Acero et al. 2011; Kitadokoro et al. 2012, accepted 2019; Kawabata et al. 2017).

Fungal cutinases (from *F. solani* and *H. insolens*) have homologous structures that are smaller than those of actinomycete cutinases. Cbotu_EstA has the largest structure among cutinase-related enzymes (Table 1), even after the N-terminal 71 residues are removed (Fig. S3).

In summary, the basic structure of PET hydrolases must incorporate the following features: the shallow open active site should be accessible to the solvent, the active cleft should have an appropriate space for bulky aromatic compounds, and the active cleft region should have affinity with hydrophobic materials. Moreover, thermostability is needed for significant degradation of the PET building block.

## The role of divalent cations in catalysis

Although only Cut190 displays both active (Ca^2+^-bound) (PDB IDs: 4WFJ and 4WFK) and inactive (Ca^2+^-free) (PDB ID: 4WFI) states (Miyakawa et al. 2015), the active state corresponds to those of Est119 and other cutinases (PDB IDs: 4EB0, 3VIS, 3WYN, 4CG1, 4CG2, 4CG3, and 5LUI, 5LUJ, 5LUK, and 5LUL) (Sulaiman et al. 2014; Kitadokoro et al. 2012; Kawai et al. 2013; Roth et al. 2014; Ribitsch et al. 2017). The activities and thermal stabilities of these cutinases increase in the presence of Ca^2+^ and Mg^2+^ (Kawai et al. 2013, 2014; Sulaiman et al. 2014; Thumarat et al. 2012; Then et al. 2015; Miyakawa et al. 2015). Est119 retains the same overall structure with or without Ca^2+^ (PDB IDs: 3WYN and 3VIS). Cutinases 1 and 2 from *T. cellulosilytica* (Thc_Cut1 and Thc_Cut2) and Thc_Cut2 mutants, either bound or not bound to Mg^2+^ or Ca^2+^ (PDB IDs: 5LUI, 5LUJ, 5LUK, and 5LUL), also retain the same overall structures. Three Ca^2+^ bind at three different sites in the mutant S176A of Cut190* (PDB ID: 5ZNO) (Numoto et al. 2018) (Fig. 3a), and their roles have been analyzed by mutation (Oda et al. 2018). Binding of Ca^2+^ at site 1 (Ser76, Ala78, Phe81, and Asn133) is unique to Cut190 (related to activation), but binding at site 2 (Glu220, Asp250, and Glu296) is common in Est119 and other cutinases (related to thermostabilization). These three amino acids at site 2 are well conserved among cutinases as shown in Fig. S2. Ca^2+^ binding at site 3 (Asp204 and Thr206) has not been reported for cutinases other than Cut190 (subsidiary to sites 1 and 2). Molecular dynamic (MD) simulations indicated that the Ca^2+^-bound structure fluctuates less than the Ca^2+^-free structure, leading to higher thermal stability (Inaba et al. 2018; Numoto et al. 2018). We recently analyzed the effect of various divalent cations (Zn^2+^, Mn^2+^, and Mg^2+^) on the activity and thermal stability of Cut190* compared with that of Ca^2+^ (Senga et al. 2019). Zn^2+^ can readily replace Ca^2+^ (Numoto et al. 2018), but the enzyme activity of Cut190* in the presence of Zn^2+^ is very low. However, the activity of Cut190* with Mn^2+^ and Mg^2+^ is maintained to some extent but it is lower than that with Ca^2+^.

Then et al. (2015) reported that Ca^2+^ and Mg^2+^ increase *T*_m_ values of *T. fusca* cutinases (TfH, BTA2, Tfu_882, TfCut1, and TfCut2) by 10.8–14.1 °C. The thermostabilities of these polyesterases are sufficient to enable degradation of PET-GF at 65 °C in the presence of 10 mM Ca^2+^ or Mg^2+^, but Ca^2+^ shows better results in general. The activity was highest with TfH, and Tf_Cut2 showed an almost identical activity to that of TfH. The binding sites of Ca^2+^ and Mg^2+^ in TfCut2 have been calculated by independent MD simulations and were the same to site 2 in Cut190*. The roles of Ca^2+^ and Mg^2+^ in Thc_Cut2 have not been elucidated yet, but they are most probably the same as those in TfCut2. Cbotu_EstA has a zinc-binding domain, and mutation of amino acids in this domain increases the activity on PET without changing the zinc-binding ability (Biundo et al. 2018a). The role of this zinc-binding domain remains to be elucidated but would appear to be different from those of metal-binding domains in cutinases.

Further studies are needed to determine whether metal ions are relevant to the reaction in other cutinases.

## Catalytic mechanism for hydrolysis of PET by cutinases

Both endo-type and exo-type hydrolases of glucans (e.g., amylases and cellulases) are known, but no exo-type hydrolase of polyesters has been reported to date. Endo-type hydrolysis has been indicated for PET because matrix-assisted laser desorption/ionization time-of-flight (MALDI-TOF) mass spectrometry of the residual insoluble materials indicated an increase in the tetramer fragment of PET (average Mw = 3500) (Eberl et al. 2009). Herrero Acero et al. (2011) suggested that PET hydrolysis is catalyzed by endo-type scission to cleave internal ester bonds using bis(benzoyloxyethyl)terephthalate (3PET). Hydrolysis products of PET, bis(hydroxyethyl)terephthalate (BHET), mono(hydroxyethyl)terephthalate (MHET), and terephthalic acid (TPA), have been identified, although the ratio of products differs based on the enzyme concentration used, incubation temperature, and time (Vertommen et al. 2005; Ronqvist et al. 2009; Wei et al. 2012; Barth et al. 2015). TPA is regarded as the final product (Vertommen et al. 2005; Eberl et al. 2009; Ronqvist et al. 2009; Barth et al. 2015; Oda et al. 2018). However, a surface erosion process is suggested for the enzymatic hydrolysis of PET (Zhang et al. 2004; Müller et al. 2005; Müller 2006; Wei et al. 2014a). The classical Michaelis–Menten enzyme kinetic model can be used for homogeneous reactions and is based on enzyme-limiting conditions. However, insoluble polymer degradation is limited to the surface of the substrate (substrate-limiting). The degradation of PET film should follow a heterogeneous kinetic model based on substrate-limiting conditions (Ronqvist et al. 2009). In contrast, Barth et al. (2015) analyzed hydrolysis of PET nanoparticles based on enzyme-limiting conditions. Mutation of Cut190* has been evaluated by the decrease in turbidity of a PBSA suspension (Kawai et al. 2014; Kawabata et al. 2017; Oda et al. 2018), which is based on enzyme-limiting conditions.

PETase showed no significant conformational changes in the main chain structure upon ligand binding, and the active site is wider than that of other cutinases (Han et al. 2018; Chen et al. 2018). These authors of this work proposed that W156 wobbling in PETase (adjacent S185 allows a space for W156 to rotate) is important for substrate binding and product release (Fig. S4b), but W155 in TfCut2 cannot wobble as the side chain of H184 stacks with the side chain of W155. However, as TfCut2 and other homologous cutinases can significantly hydrolyze PET-GF, there is no doubt that their active sites can efficiently bind the substrate. Numoto et al. (2018) indicated that no movement of W201 in Cut190* (corresponding to W156 in PETase and W155 in TfCut2) is observed, but the opposite amino acids, F106 and T107, derive different conformations upon substrate binding (Fig. S4a). Several other differences were found between PETase and Cut190*. PETase shows no significant structural differences on substrate docking, except for the movement of W156, but the β1–β2, β3–α2, and β4–α3 loops of Cut190* derive different conformations upon substrate docking and reaction product release. The benzene ring of HEMT is T-stacked to an aromatic ring of W156 of PETase but not to that of Y58 because benzene rings of W156 and of Y58 are not parallel (PDB ID: 5XH3). In contrast, the aromatic rings of W201 and F106 are parallel in Cut190* complexed with model substrates (PDB IDs: 5ZRR and 5ZRS) (Fig. S4a). As predicted in the 3D docking structure of Cut190* with a PET model compound (TETETET, where T and E are terephthalate and ethylene glycol units, respectively) (Kawabata et al. 2017), a benzene ring would be T-stacked to W201 and F106 and then M177 would support a benzene ring from below. In either case, fluctuation of aromatic amino acids is considered important for fixing a benzene ring of PET by T stacking. In addition, a short tunnel covering an active site is formed in Cut190* (Numoto et al. 2018). The tunnel structure has also been suggested for the reaction mechanism of exo-cellulases, wherein cellobiohydrolase slides on a cellulose molecule by hydrolyzing it to cellobiose (Divne et al. 1994). Whether a similar mechanism is observed in Cut190* has yet to be elucidated in detail. Cut190* may be adsorbed on a loose polymer chain on the surface and may hydrolyze an ester bond, releasing the carboxylic end of the TPA portion and the hydroxyl end of the ethylene glycol portion. Furthermore, one possible mechanism is the detachment of Cut190* from the polymer chain to attach onto a new polymer chain (route 1). Another possible mechanism is that Cut190* stays and slides on the polymer chain (route 2) while keeping a short tunnel and forms a new tetrahedron structure necessary for the catalysis, which must be continued until arriving at the end of a polymer chain or the crystalline region, and then forms an open structure to accept a new polymer chain. The proposed routes (1 and 2) are illustrated in Fig. S5.

## Prospective applications of PET hydrolases in PET waste management and other fields

The majority of plastics are disposed of within 1 year after manufacture. Of these, 14% of packaging materials are currently collected for recycling and another 14% are incinerated for energy recovery, but 40% of plastics are landfilled and 32% escape the collection system (Ellen MacArthur Foundation and World Economic Forum, 2014; http://www3.weforum.org/docs/WEF_The_New_Plastics_Economy.pdf). Plastic waste that escapes the collection system enters natural environments—on land and in oceans. Plastics already threaten wildlife and are entering the food chain as microplastics. The reduction in the use of plastics and the replacement of petroleum-based plastics with natural materials and bio-based plastics are required and planned. The development of recycling systems must be established concurrently.

Because of its excellent physical and chemical properties, PET has various applications in textiles, packaging materials, and bottles. PET bottles are systematically collected and can be recycled as bottles and polyester fibers. To stop the limitless increase in production and to promote recovery, recycling of used PET bottles should be simple and requires urgent countermeasures. A Japanese company (TEIJIN) practiced the recycling of polyester fibers and PET bottles under the project “ECOCIRCLE” since 2002, with the recycled PET being exported to China. However, this project was stopped in 2018 because the Chinese government announced a ban on importation of materials produced from plastic waste in 2017, and trading of used PET bottles (the raw material for the recycling) became uneconomical. On January 17, 2019, CARBIOS (https://carbios.fr/) and Toulouse White Biotechnology (TWB) announced the receipt of EUR 7.5 million funding, over a period of 39 months, from the Investments for the Future program operated by ADEME (the French Environment and Energy Management Agency) to accelerate the industrialization of the bio-recycling of PET plastics and fibers. Chemical recycling of PET waste produced during the production process can be practiced in situ. The major problems are recycling and appropriate treatment of PET waste after use. Plastic recycling is categorized into three types: thermal recycling (used as fuels), material recycling (melted and reused once), and chemical recycling (converted to raw materials and used for synthesis, which can be repeated). The most desirable type is chemical recycling, for which PET hydrolase can be used. As described above, enzymatic hydrolysis cannot yet be applied to bottles and fibers, although every researcher in this field seeks to apply their enzymes to any PET waste, including tough materials such as bottles and fibers. However, research for some enzymes has already reached practical levels for their application to amorphous PET films used in packaging (Wei and Zimmermann 2017).

Polymeric materials with high polymerization degrees (e.g., 100) have rather different physicochemical properties from those with low polymerization degrees (e.g., 10–50 carbon atoms), and the latter can be taken up by a cell for further metabolization (Lucas et al. 2008; Restrepo-Flórez et al. 2014). Depolymerized PET up to 10–50 carbon atoms is likely to be susceptible to attack by PET hydrolases to form TPA and ethylene glycol, as 3PET and cyclic trimers that are readily hydrolyzed by carboxylesterases (Billig et al. 2010; Eberl et al. 2009; Figueroa et al. 2006; Herrero Acero et al. 2011; Ribitsch et al. 2012a, b). In addition, micronization of PET materials markedly improves their subsequent biodegradation by increasing the accessible surface area for polyesterases (Gamerith et al. 2016 and 2017). Therefore, chemical or physical pretreatment of plastic waste may be required in biocatalytic recycling (Wei and Zimmermann 2017). Appropriate pretreatment would promote the efficiency of enzymatic recycling of PET. Donelli et al. (2010) compared the surface structural changes induced on PET films by alkali and cutinase treatments and observed that both treatments induced structural and conformational rearrangements with a consequent increase in crystallinity. Quartinello et al. (2017) depolymerized PET fiber (at 250 °C and 39 bar) to a powder consisting of 85% TPA, and the remaining oligomers were subjected to enzymatic hydrolysis by a cutinase, finally yielding TPA with 97% purity that is comparable to synthesis grade TPA (98% purity).

As described in the “Introduction,” microplastics are already found in the intestines of wild animals, indicating that microplastics could be ingested by humans via the food chain. Microplastics are produced from plastic waste left in the environment and from wastewater treatment plants. For the latter source, one approach to improve polymer degradation in wastewater would utilize polymer-degrading enzymes and their microbial producers (Haernvall et al. 2018). Synthetic polyesters are the second largest class of ingredients in household products that enter sewage plants. Polyesterases must have broad substrate specificities to be useful for sewage treatment. This kind of application may cause a serious controversy as mutants are more effective than the wild type, and should be cloned in appropriate hosts. Therefore, release of the recombinant microbes and safety of the ecosystem must be carefully evaluated and balanced.

Cut190, Est119, and Est1 have broad substrate specificity toward various polyesters, aliphatic, aliphatic-*co*-aromatic, and PET. BTA hydrolase was originally expected to hydrolyze BTA, but PET hydrolytic activity was confirmed later (Kleeberg et al. 2005; Müller et al. 2005). Broad substrate specificities are generally expected for actinomycete cutinases. In other words, these cutinases can be used for polyester recycling of aliphatic and aliphatic-*co*-aromatic polyesters, and amorphous PET. For example, vessels or containers made of biodegradable/bio-based materials do not need to be fractionated as they are subjected to enzymatic digestion by these PET hydrolases. Biodegradable/bio-based polymers are not currently targeted for recycling, but their waste treatment after use will become an environmental issue in the future. Polyesterases with broad substrate specificities are more desirable than those with substrate specificities for certain materials. PET hydrolases can be applied for the functionalization of general polyesters in addition to PET, which displays carboxyl and hydroxyl groups on the surface, without damaging the bulk properties of polyesters, and they may be useful for forming a complex structure with chemicals.

As emphasized in this review, PET hydrolase must be thermostable, not only because PET hydrolysis needs a higher temperature than the *T*_g_ value (preferably 65–70 °C in aqueous solution) but also because the degradation of other polyesters is promoted at higher temperatures. In addition, high temperatures over 55 °C contribute to the disinfection of digested plastic waste. Thermostable enzymes are beneficial for applications because catalysis increases at elevated temperatures and the biocatalysts can be maintained for a long time at ambient temperatures. PET hydrolases are cutinases, and their application to various fields is already well known (Carvalho et al. 1999; Bornscheuer 2002; Pio and Macedo 2009; Chen et al. 2013; Nyyssölä 2015). Hence, thermostable enzymes are desirable.

Finally, it should be noted that research on PET hydrolases is also contributing new advances in biocatalysis research. Enzyme reactions are, in general, good at attacking water-soluble or liquid materials, but polyesterases must attack solid materials. For the hydrolysis of PET, we need to understand the properties of PET materials and the structures of PET hydrolases. For PET, enzymatic kinetics do not follow the classical Michaelis–Menten model for homogeneous reactions but necessitate a different kinetic model for heterogeneous reactions.

## Electronic supplementary material

ESM 1 (PDF 1132 kb)
