# Supplementary material for: Current knowledge on enzymatic PET degradation and its possible application to waste stream management and other fields
Source: Appl Microbiol Biotechnol. 2019 Apr 8;103(11):4253–68. doi: 10.1007/s00253-019-09717-y (PMC6505623; doi:10.1007/s00253-019-09717-y)
Supplement: Supplementary file 1 — (PDF 1132 kb) [file 253_2019_9717_MOESM1_ESM.pdf]

Mini-review for Applied Microbiology and Biotechnology

Supporting information

Current knowledge on enzymatic PET degradation and its possible application to design waste stream management and other applications

Fusako Kawai,[1,4*] Takeshi Kawabata,[2] Masayuki Oda[3]

[1]Center for Fiber and Textile Science, Kyoto Institute of Technology, 1 Hashigami-cho, Matsugasaki, Sakyo-ku, Kyoto, Kyoto 606-8585, Japan

[2]Institute for Protein Research, Osaka University, 3-2 Yamadaoka, Suita, Osaka 565-0871, Japan

[3]Graduate School of Life and Environmental Sciences, Kyoto Prefectural University, 1-5 Hangi-cho, Shimogamo, Sakyo-ku, Kyoto, Kyoto 606-8522, Japan

[4]Present address: Emeritus Professor, Okayama University, Japan

[*]Corresponding author: Fusako Kawai

E-mail: fkawai@kit.ac.jp. Tel and Fax: +81-75-703-8576.

Table S1. Reaction products from PET-GF, PET-S and PET microfiber at 30 °C.

The incubations were performed in a reaction mixture (1 ml) including Cut190*(Q138A/D250C-E296C), 2 μM; $CaCl_2$, 2.5 mM; glycerol, 24%; HEPES buffer (pH 8.5), 100 mM; and each PET (the same as previously described; Oda et al. 2018) under shaking in a water bath at 30 °C. At intervals, an aliquot of the reaction mixture was withdrawn, the absorbance of which was measured at 240 nm ($\varepsilon = 1.38 \times 10^4$) (Oda et al. 2018).

Incubation time at 30 °C (h)	Products								
	PET-GF			PET-S			Microfiber		
	μmole/ml	μmole/both sides of 6mmΦ	degradation %[a]	μmole/ml	μmole/both sides of 6mmΦ	degradation %[b]	μmole/ml	μmole/as both sides of 6mmΦ	degradation %[c]
22	0.015	0.015	0.03	0.013	0.013	0.01	0.13	0.037	2.3
44	0.023	0.023	0.05	0.016	0.016	0.01	0.42	0.12	7.4
66	0.027	0.027	0.06	0.014	0.014	0.01	0.84	0.24	14.8
93	0.027	0.027	0.06	0.014	0.014	0.01	1.25	0.35	22.0
113	0.041	0.041	0.09	0.013	0.013	0.01	1.41	0.40	24.8

[a] calculated against 8.4 mg (43.8 μmoles as MHET unit; crystallinity of 6.3%; 6mmΦ×0.25mm-tchick).
[b] calculated against 22.7 mg (118 μmoles as MHET unit; crystallinity of 8.4%; 6mmΦ×0.6mm-tchick).
[c] calculated against 1.1 mg (5.72 μmoles as MHET unit; crystallinity of 14.1%; approximately 1cm-square).

The amounts of products from PET-GF and PET-S were almost kept constant in 22-113 h at approximately 0.02-0.04 and 0.01-0.02 μmole/both side of 6mmΦ, respectively. On the other hand, hydrolysis of microfiber PET increased with increased reaction time, finally reaching 24.8% of degradation in 113 h. Degradation of microfiber is approximately ten times and more as much as those of PET-GF and PET-S, although crystallinities of PET-GF and PET-S are approximately 45% and 60% of the value of microfiber, respectively. These results indicate that orientation/surface topology of microfiber is quite different (no orientation) from those of two films, which are supported by that microfiber absorbed a lot of water, resulting in the approximately duplicate width after incubation, as shown below. In addition, hydrolysis of PET films is limited at 30 °C, corresponding to the surface hydrophilization level, although microfiber is hydrolyzed by the surface erosion level even at 30 °C.

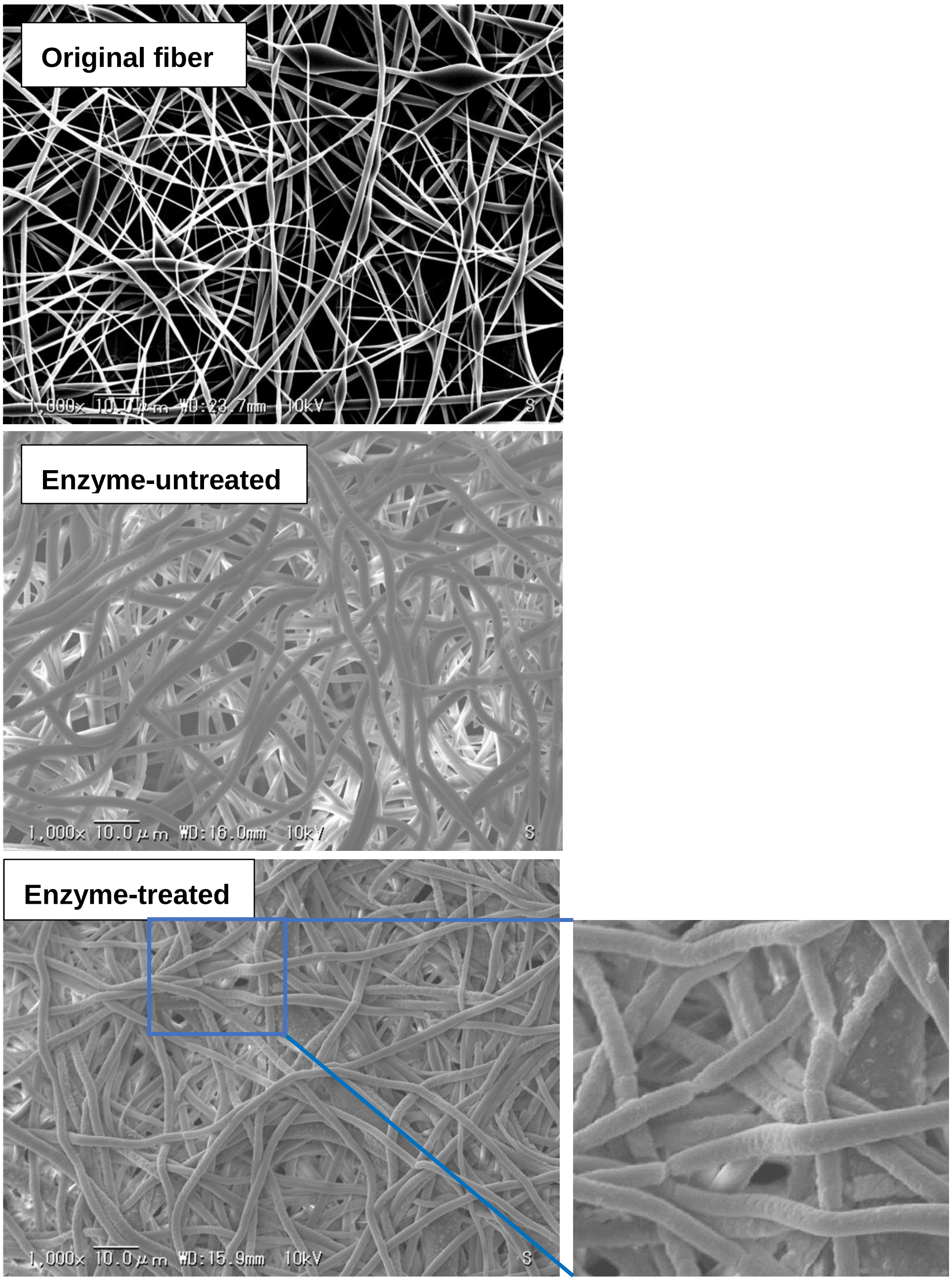

Enzyme reactions and SEM observation were performed as described previously (Oda et al. 2018).

Table S2. Comparison of degradation abilities of PETase and Cut190* toward PET films

Enzyme used	PET film (crystallinity)	Film thickness	Film weight	unit (MHET) in the film	Total products in a reaction mixture	Degradation rate (%)	Products/cm^2
PETase[a]	Synthesized[a] (1.9%)	0.2 mm	7.57 mg	39.4 μmoles	0.09 μmoles/300 μl	0.23	0.159 μmoles
Cut190*[b] (Cut190* mutant)[b]	PET-GF[b] (6.3%)	0.25 mm	8.40 mg	43.7 μmoles	7.01 μmoles/ml (14.7 μmoles/ml)	16.0 ± 1.4 (33.6 ± 3.0)	12.3 ± 1.2 (28.6 ± 2.3)
Cut190*[b]	PET-S[b] (8.4%)	0.6 mm	22.7 mg	118 μmoles	29.2 μmoles/ml	59.2 ± 2.1[c]	46.9 ± 1.6

[a] Data were calculated from Yoshida et al. 2016. PET film (0.2 mm-thick, 6mmΦ: 56.52 mm^2 as both sides; density, 1.3378 g/cm^3) was incubated in 300 μl with 50 nM PETase in pH 7.0 buffer for 18 h at 30 °C. Total amount of products was shown in Fig. 2D (b) as approximately 0.3 mM.

[b] PET-GF and PET-S used are the same as previously described (Oda et al. 2018). The average weight of 6 mmΦ PET-GF was 8.4 mg. The weight of PET-S was calculated as 22.7 mg. The incubations were performed with Cut190* at 65 °C for PET-GF (70 °C with the Cut190* mutant) and at 60 °C for PET-S. The values were cited from Table 3 in Oda et al. 2018.

[c] Calculated as 0.25 mm-thick.

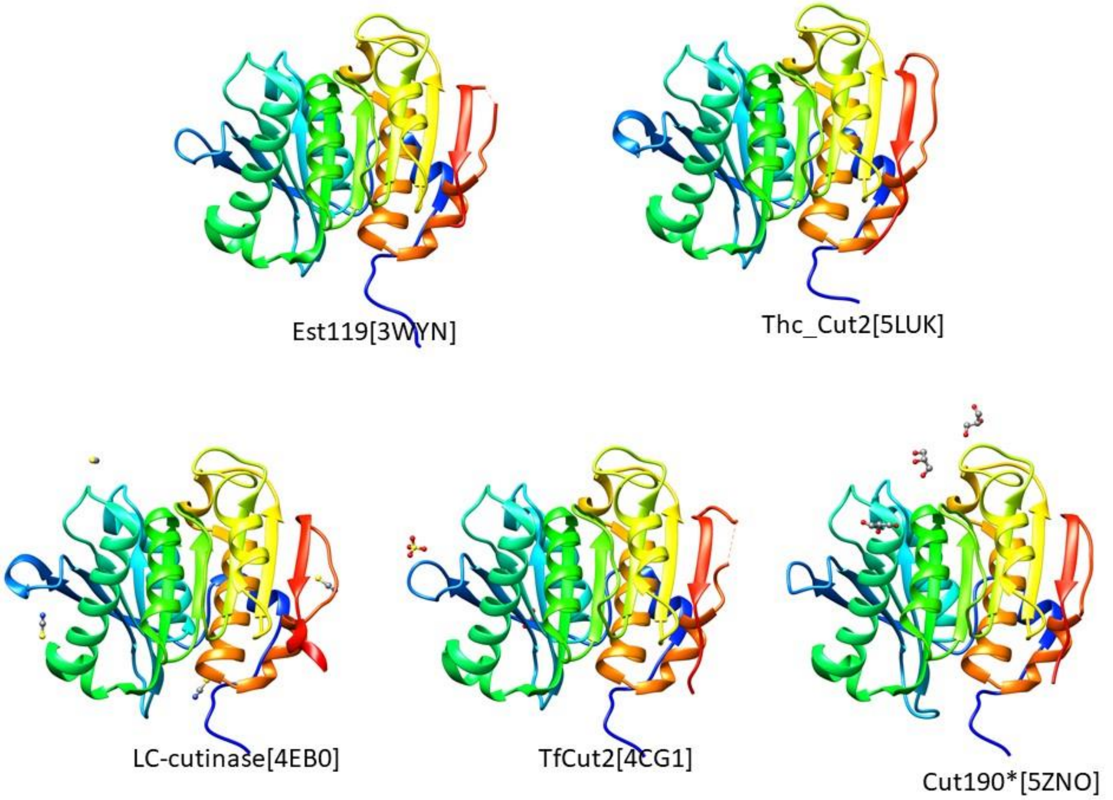

Figure S1. Crystal structures of PET hydrolases and homologous enzymes from actinomycetes. PDB IDs are shown in blackets.

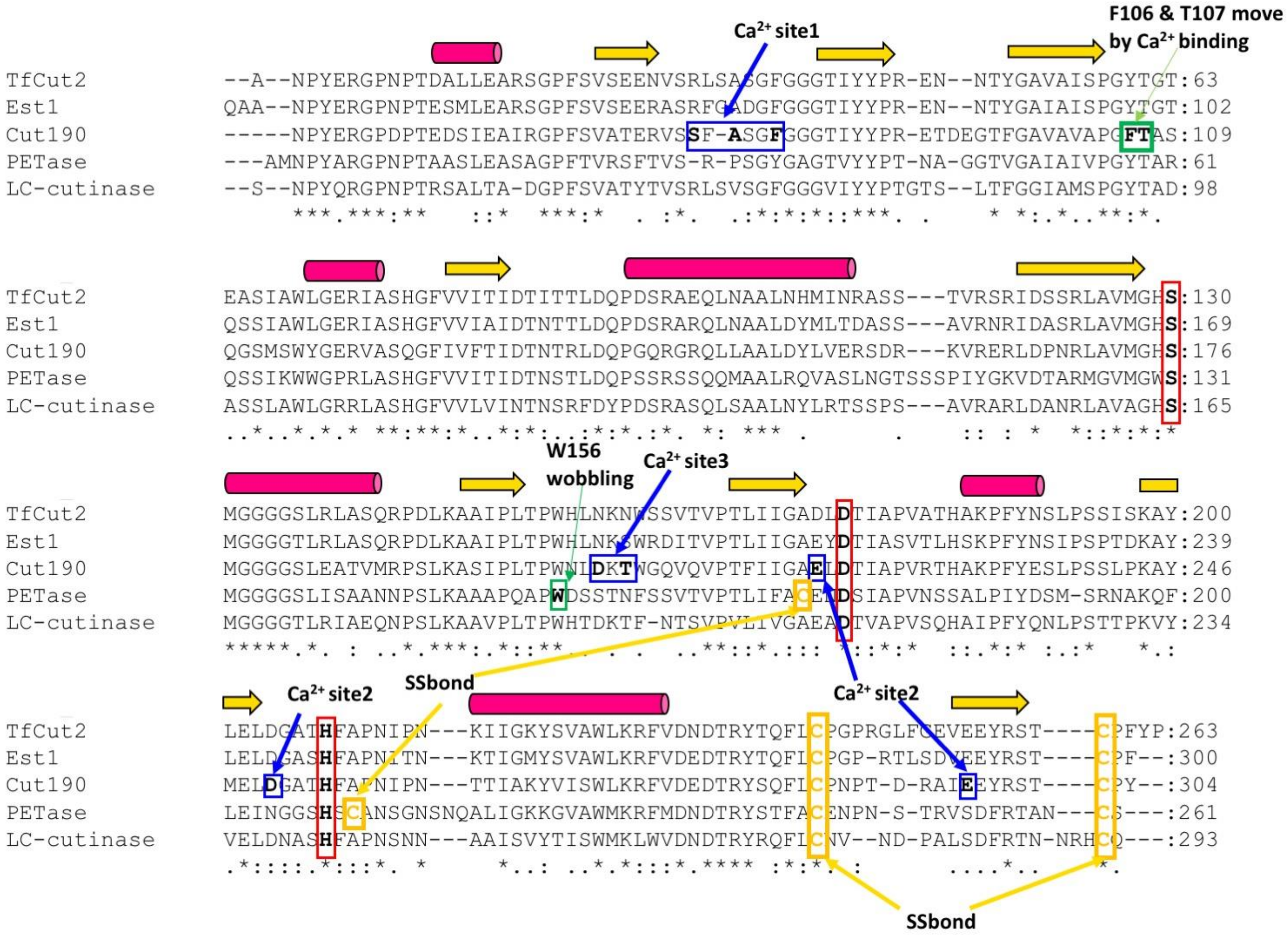
Ca^{2+} site1
F106 & T107 move
by Ca^{2+} binding
TfCut2 --A--NPYERGPNPTDALLEARSGPFSVSEENVSRLSASGFGGGTIYYPR-EN--NTYGAVAISPGYTGT:63
Est1 QAA--NPYERGPNPTESMLEARSGPFSVSEERASRFGADGFGGGTIYYPR-EN--NTYGAIAISPGYTGT:102
Cut190 -----NPYERGPDPTEDSIEAIRGPFSVATERVSSF-ASGFGGGTIYYPR-ETDEGTFGAVAVAPGFTAS:109
PETase ---AMNPYARGPNPTAASLEASAGPFTVRSFTVS-R-PSGYGAGTVYYPT-NA-GGTVGAIAIVPGYTAR:61
LC-cutinase --S--NPYQRGPNPTRSALTA-DGPFSVATYTVSRLSVSGFGGGVIYYPTGTS--LTFGGIAMSPGYTAD:98
TfCut2 EASIAWLGERIASHGFVVITIDTITTLDQPDSRAEQLNAALNHMINRASS---TVRSRIDSSRLAVMGHS:130
Est1 QSSIAWLGERIASHGFVVIAIDTNTTLDQPDSRARQLNAALDYMLTDASS---AVRNRIDASRLAVMGHS:169
Cut190 QGSMSWYGERVASQGFIVFTIDTNTRLDQPGQRGRQLLAALDYLVERSDR---KVRERLDPNRLAVMGHS:176
PETase QSSIKWWGPRLASHGFVVITIDTNSTLDQPSSRSSQQMAALRQVASLNGTSSSPIYGKVDTARMGVMGWS:131
LC-cutinase ASSLAWLGRRLASHGFVVLVINTNSRFDYPDSRASQLSAALNYLRTSSPS---AVRARLDANRLAVAGHS:165
W156
wobbling
Ca^{2+} site3
TfCut2 MGGGGSLRLASQRPDLKAAIPLTPWHLNKNWSSVTVPTLIIGADLDTIAPVATHAKPFYNSLPSSISKAY:200
Est1 MGGGGTLRLASQRPDLKAAIPLTPWHLNKSWRDITVPTLIIGAEYDTIASVTLHSKPFYNSIPSPTDKAY:239
Cut190 MGGGGSLEATVMRPSLKASIPLTPWNLDKTWGQVQVPTFIIGAELDTIAPVRTHAKPFYESLPSSLPKAY:246
PETase MGGGGSLISAANNPSLKAAAPQAPWDSSTNFSSVTVPTLIFACENDSIAPVNSSALPIYDSM-SRNAKQF:200
LC-cutinase MGGGGTLRIAEQNPSLKAAVPLTPWHTDKTF-NTSVPVLIVGAEADTVAPVSQHAIPFYQNLPSTTPKVY:234
Ca^{2+} site2
SSbond
Ca^{2+} site2
TfCut2 LELDGATHFAPNIPN---KIIGKYSVAWLKRFVDNDTRYTQFLCPGPRGLFGEVEEYRST----CPFYP:263
Est1 LELDGASHFAPNITN---KTIGMYSVAWLKRFVDEDTRYTQFLCPGP-RTLSDWEEYRST----CPF--:300
Cut190 MELDGATHFAPNIPN---TTIAKYVISWLKRFVDEDTRYSQFLCPNPT-D-RAIEEYRST----CPY--:304
PETase LEINGGSHSCANSGNSNQALIGKKGVAWMKRFMDNDTRYSTFACENPN-S-TRVSDFRTAN---CS---:261
LC-cutinase VELDNASHFAPNSNN---AAISVYTISWMKLWVDNDTRYRQFLCNV--ND-PALSDFRTN-NRHCQ---:293
SSbond

Figure S2. Multiple 3D structural alignment of PET hydrolases. The program MATRAS was employed to generates 3D alignments (Kawabata 2003). The positions of C-terminal cysteine were manually modified. The consensus secondary structures are shown on the top of the alignment, conserved sites are shown as asterisks on the bottom. Following PDB IDs are used for the alignment: 4CG1 for TfCut2, 3VIS for Est1, 4WFK for Cut190, 5XG0 for PETase and 4EB0 for LC-cutinase. Amino acids comprising the catalytic triad are boxed in red.

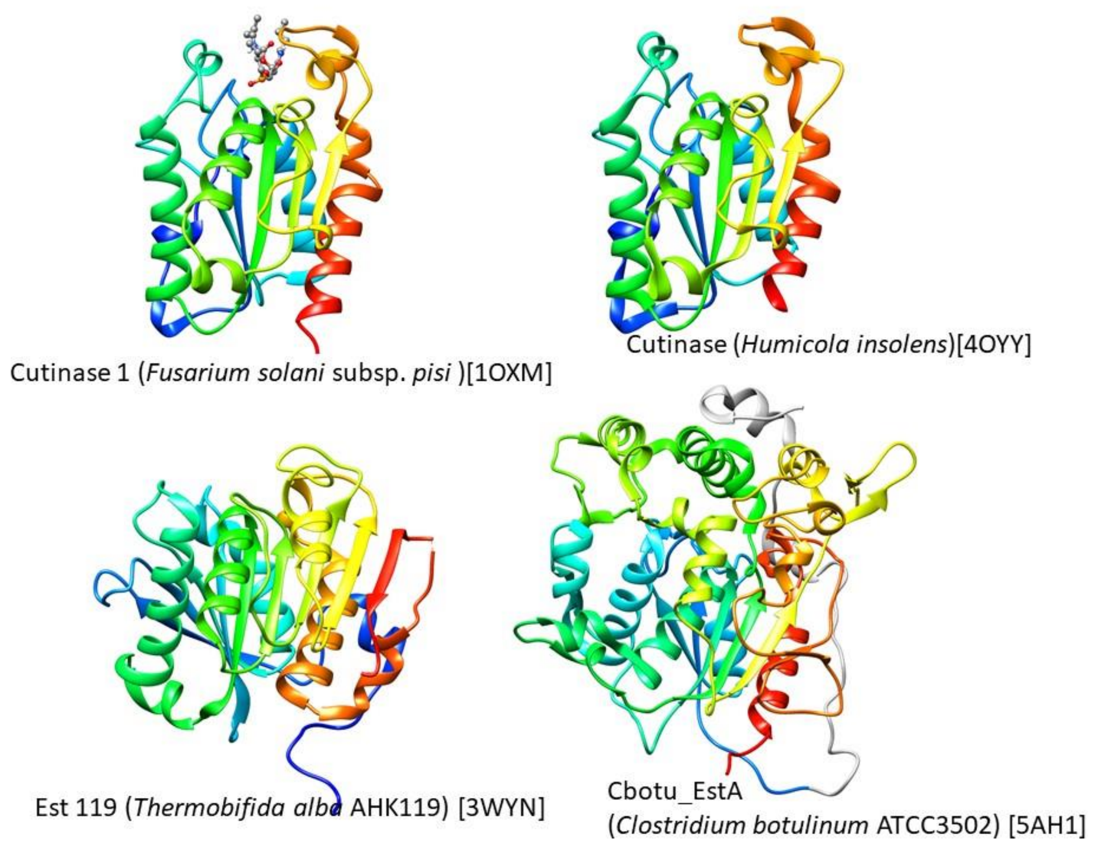

Figure S3. Comparison of cutinase-related structures. The truncated N-terminal 71 residues of Cbotu_EstA are shown in gray. Strain names are shown in parentheses and PDB IDs are shown in blackets.

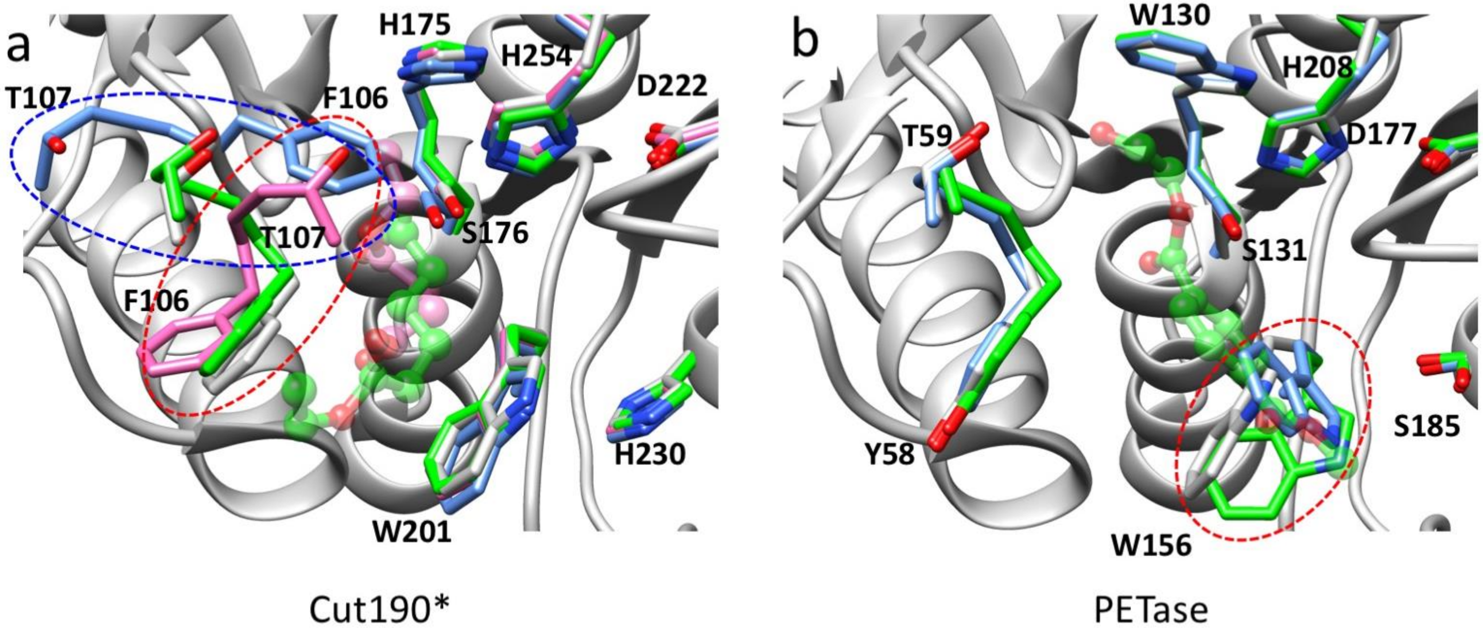

Figure S4. Comparison of catalytic site 3D structures of Cut190* and PETase. **a** Four structures of Cut190*. The structures are colored as follows: 4WFK (apo structure with Ca^{2+} on site1) in gray, 4WFI (apo structure without Ca^{2+}) in blue, 5ZRR(with ethyl succinate and four Zn^{2+}) in pink, and 5ZRS (with ethyl adipate, Ca^{2+} on site1 and three Zn^{2+}) in green. The substrates (ethyl succinate and ethyl adipate) are displayed as transparent ball & stick models. Positions of F106 and T106 are quite different among the structures. **b** Three structures of PETase. The structures are colored as follows: 5XJH (apo structure) in gray, 5XG0(apo structure) in blue, and 5XH3 (with HEMT) in green. The substrate HEMT is displayed as a transparent model. Sidechain conformations of W156 are wobbling among the structures.

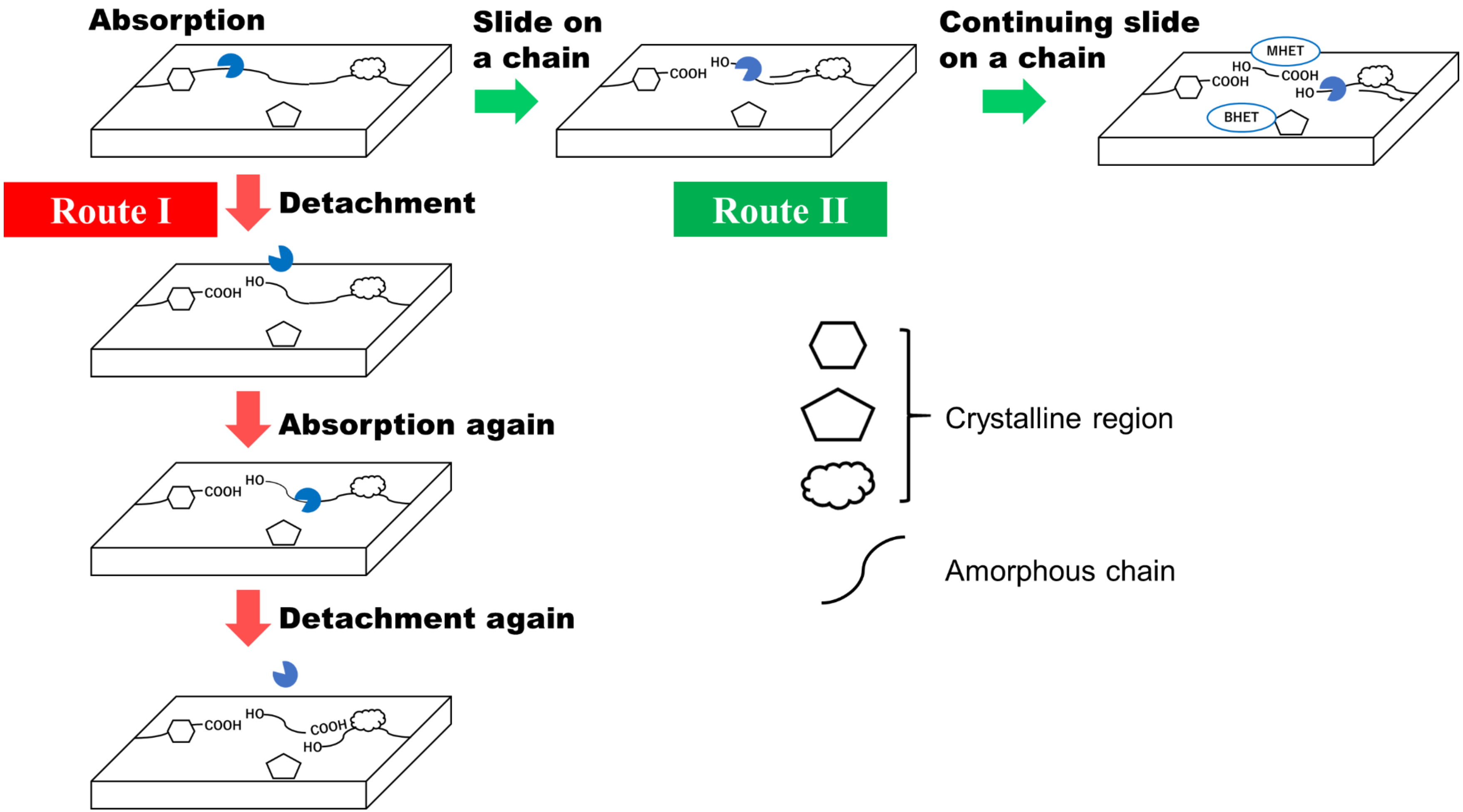

Figure S5. Presumed mechanism of PET hydrolysis by Cut190*

References

Kawabata T (2003) MATRAS: a program for protein 3D structure comparison. Nucleic Acids Res **31**:3367-3369

Oda M, Yamagami Y, Inaba S, Oida T, Yamamot M, Kitajima S, Kawai F (2018) Enzymatic hydrolysis of PET: functional roles of three Ca^{2+} ions bound to a cutinase-like enzyme, Cut190*, and its engineering for improved activity. Appl Microbiol Biotechnol **102**: 10067-10077

Yoshida S, Hiraga K, Takehana T, Taniguchi I, Yamaji H, Maeda Y, Toyohara K, Miyamoto K, Oda K (2016) A bacterium that degrades and assimilates poly(ethylene terephthalate). Science **351**: 1196-1199
